# Varied distributions of late gadolinium enhancement found among patients meeting cardiovascular magnetic resonance criteria for isolated left ventricular non-compaction

**DOI:** 10.1186/1532-429X-15-20

**Published:** 2013-02-20

**Authors:** Junyi Wan, Shihua Zhao, Huaibing Cheng, Minjie Lu, Shiliang Jiang, Gang Yin, Xiaojin Gao, Yuejin Yang

**Affiliations:** 1 Department of Radiology, State Key Laboratory of Cardiovascular Disease, Fuwai Hospital, National Center for Cardiovascular Diseases, Chinese Academy of Medical Sciences and Peking Union Medical College, Beijing 100037, People’s Republic of China; 2 Department of Cardiology, State Key Laboratory of Cardiovascular Disease, Fuwai Hospital, National Center for Cardiovascular Diseases, Chinese Academy of Medical Sciences and Peking Union Medical College, Beijing, 100037, People’s Republic of China

**Keywords:** Cardiomyopathy, Cardiac magnetic resonance, Late gadolinium enhancement

## Abstract

**Background:**

Late gadolinium enhancement (LGE) is identified frequently in LVNC. However, the features of this findings are limited. The purpose of the present study was to describe the frequency and distribution of LGE in patients meeting criteria for left ventricular non-compaction (LVNC), as assessed by cardiovascular magnetic resonance (CMR).

**Methods:**

Forty-seven patients (37 males and 10 females; mean age, 39 ± 18 years) considered to meet standard CMR criteria for LVNC were studied. The LGE images were obtained 15 ± 5 min after the injection of 0.2 mmol/kg of gadolinium-DTPA using an inversion-recovery sequence, and analyzed using a 17-segment model.

**Results:**

Mean number of non-compacted segments per patient was 7.4 ± 2.5 and the NC:C was 3.2 ± 0.7. Non-compaction was most commonly noted in the apical segments in all patients. LGE was present in 19 of the 47 patients (40%), and most often located in the ventricular septum. The distribution of LGE was subendocardial (n = 5; 6%), mid-myocardial (n = 61; 68%), subepicardial (n = 10; 11%), and transmural (n = 14; 15%) in total of 90 LGE (+) segments.

**Conclusions:**

In patients considered to meet criteria for LVNC, LGE distributions visible were strikingly heterogeneous with appearances potentially attributable to three or more distinct cardiomyopathic processes. This may be in keeping with previous suggestions that the criteria may be of low specificity. Further work is needed to determine whether conditions such as dilated cardiomyopathy, previous myocardidtis or ischaemic heart disease increase the apparent depth of non-compact relative to compact myocardium.

## Background

Left ventricular non-compaction (LVNC) is a disorder characterized by numerous prominent ventricular trabeculations and deep intratrabecular recesses [1], high mortality and morbidity rates from heart failure, systemic thromboemboli, and malignant ventricular arrhythmias [2-6]. It can occur in isolation or in association with congenital cardiac malformations, genetic syndromes, or neuromuscular disorder. Echocardiography is usually the initial cardiac imaging modality used to characterize myocardial pathologies [7-9]. Recently, cardiovascular magnetic resonance (CMR) has been increasingly used to describe the morphological appearance of the ventricular non-compaction [10-13]. In addition, late gadolinium-enhanced CMR has been found to be useful for detecting myocardial fibrosis. However, the descriptions of late gadolinium enhancement (LGE) features are limited in these reports.

The purpose of this retrospective study is to describe the frequency and distribution of LGE in patients considered to meet CMR cine imaging criteria for LVNC.

## Methods

### Study patients

The study was conducted between April 2006 and January 2011 in our hospital. We enrolled 47 consecutive LVNC patients with first a diagnosis of LVNC. The diagnosis of LVNC was based on the presence of the following CMR and clinical criteria [14]: (a) appearance of 2 distinct myocardial layers; (b) prominent myocardial trabeculations and deep intertrabecular recesses communicating with the left ventricular cavity; (c) end-diastolic ratio of non-compacted-to-compacted(NC:C) myocardium >2.3:1, and (d) absence of other known co-existing cardiac abnormalities. Coronary artery disease (>50% diameter luminal stenosis in any of the major coronary arteries or the major coronary artery branches) was excluded by invasive coronary angiography in some but not all patients, according to the clinical judgment at the time of diagnosis by the clinical physician in this study. All patients gave informed consent, and the study was approved by the institutional ethics committee.

### CMR protocol

All CMR exams were performed using a 1.5-T unit (Avanto; Siemens Healthcare, Erlangen, Germany) with a high-performance gradient system (maximum gradient amplitude, 45 mT/m; maximum slew rate, 200 mT/m/ms). Twelve-element matrix coils (6 anterior and 6 posterior) equipped with the scanner and wireless vector electrocardiograph gating triggering were activated for data acquisition. All imaging acquisitions were captured under breath control. Scout transversal and sagittal images were acquired followed by a half-Fourier acquisition single shot turbo spin echo sequence (HASTE: repetition time/echo time [TR/TE] = 700/42 ms; voxel size = 2.5 × 1.5 × 6.0 mm; flip angle = 160°) for the exact determination of the long-axis, 4-chamber, and short-axis plane position. Retrospective electrocardiographic gating cine images were acquired in three long-axis views (LV two-chamber and four-chamber long-axis, and LV outflow tract) and a contiguous set of short-axis sections encompassing the entire LV using true fast imaging with steady-state free precession (TrueFISP: TR/TE = 40.0/1.1 ms; voxel size = 2.0 × 2.0 × 6.0 mm; flip angle = 62°). Fifteen ± 5 min after the injection of 0.2 mmol/kg of gadolinium-DTPA (Magnevist; Schering, Berlin, Germany) with an inversion-recovery sequence, the LGE images were obtained in a standard short axis view covering the entire ventricle, and in long axis views (horizontal and vertical long-axis and LV outflow tract).

### CMR analysis

All CMR data were transferred to a workstation (Siemens Medical Systems) for analysis. The LV end-diastolic diameter, LV end-diastolic volume, LV end-systolic volume, and LV ejection fraction were calculated from the short axis cine images. The NC:C myocardium in diastole was calculated for each of the three long-axis views, and only the maximal ratio was used for analysis.

The presence or absence of LGE was qualitatively determined for each LV myocardial segment using the 17 segments model, according to the American Heart Association recommendation [15] by reviewing all short and long axis contrast-enhanced images. Patterns of LGE were defined as subendocardial, subepicardial, mid-myocardial, or transmural (≥75% of any segmental wall thickness) on visual analysis by a consensus of 2 independent observers.

### Statistical analysis

All values are expressed as the mean ± SD or counts (percentage). Clinical and CMR characteristics were compared using a Student’s *t*-test for continuous variables and a chi-square test or Fisher exact test for non-continuous variables in two groups. A two-tailed P-value <0.05 was considered statistically significant. Statistical analysis was performed using the SPSS software package (SPSS 16.0; SPSS, Inc., Chicago, IL, USA).

## Results

### Patient characteristics

The clinical characteristics of the entire group are summarized in Table 1. Forty-seven patients were considered to fulfil the imaging criteria of LVNC; the mean age was 39 ± 18 years (range, 13–78 years) and 37 (79%) were male. Of the 47 patients, 42 (89%) were severely symptomatic (New York Heart Association [NYHA] functional classes III and IV). All (n = 19; 100%) LGE (+) patients were in NYHA functional class III/IV. Comparing to LGE (-) patients, a non-significant trend towards an increasing percentage (n = 23; 82%) was observed (P = 0.07). In addition, LGE (+) patients had non-significantly lower LVEF, when compared with LGE (-) patients (23 ± 8 vs. 29 ± 15%; P = 0.06). Over 24-h ambulatory Holter ECG, premature ventricular contractions (PVCs) and non-sustained ventricular tachycardia (NSVT) were significantly more common in LGE (+) patients (PVCs: 79% vs. 29%; p = 0.001; and NSVT: 47% vs.7%; p = 0.003). The mean values of LV end-diastolic diameter, LV volumes, mass, number of non-compacted segments, and non-compacted/compacted ratio between the two groups was not statistically significant.

**Table 1:** Clinical and cardiac magnetic resonance imaging characteristics of 47 patients with isolated ventricular non-compaction

	**Total patients (n =47)**	**LGE status**		
		**LGE(+) (n =19)**	**LGE(-) (n =28)**	**P-value**
*Age (yrs)*	39 ± 18	43 ± 14	36 ± 20	0.16
*Gender male, n (%)*	37 (79%)	13 (68%)	24 (86%)	0.16
*Weight (kg)*	67 ± 16	68 ± 16	66 ± 15	0.65
*NYHA functional class III/IV*	42 (89%)	19 (100%)	23 (82%)	0.07
*Symptoms*				
*Chest pain, n (%)*	5 (11%)	2 (11%)	3 (11%)	0.98
*Syncope, n (%)*	4 (9%)	2 (11%)	2 (7%)	0.68
*Systemic thrombo-embolism*	3(6%)	1 (5%)	2 (7%)	0.80
*Electrocardiogram*				
*Left bundle branch block*	7 (15%)	3 (16%)	4 (14%)	0.89
*Right bundle branch block*	5 (11%)	2 (11%)	3 (11%)	0.98
*Atrial fibrillation*	10 (21%)	4 (21%)	6 (21%)	0.98
*24 h ambulatory Holter ECG*				
*PVCs*	49%	79%	29%	0.001
*NSVT*	23%	47%	7%	0.003
*Non-compacted segments per patient*	7.4 ± 2.5	6.9 ± 1.9	7.8 ± 2.7	0.26
*Non-compacted/compacted ratio*	3.2 ± 0.7	3.0 ± 0.6	3.4 ± 0.7	0.09
*CMR Parameters*				
*LV end-diastolic diameter (mm)*	69 ± 10	69 ± 9	69 ± 11	0.92
*LV end-diastolic volume (ml)*	243 ± 97	250 ± 90	239 ± 103	0.67
*LV end-systolic volume (ml)*	189 ± 94	198 ± 88	179 ± 99	0.50
*LV ejection fraction (%)*	27 ± 13	23 ± 8	29 ± 15	0.06
*LV mass (g)*	124 ± 44	135 ± 50	117 ± 38	0.17

Data are expressed as mean ± SD. or as number of patients (percentage). *NYHAn *New York Heart Association. *PVCs *premature ventricular contractions. *NSVT *non-sustained ventricular tachycardia. *LV* left ventricular.

### CMR findings

**Figure 1:**
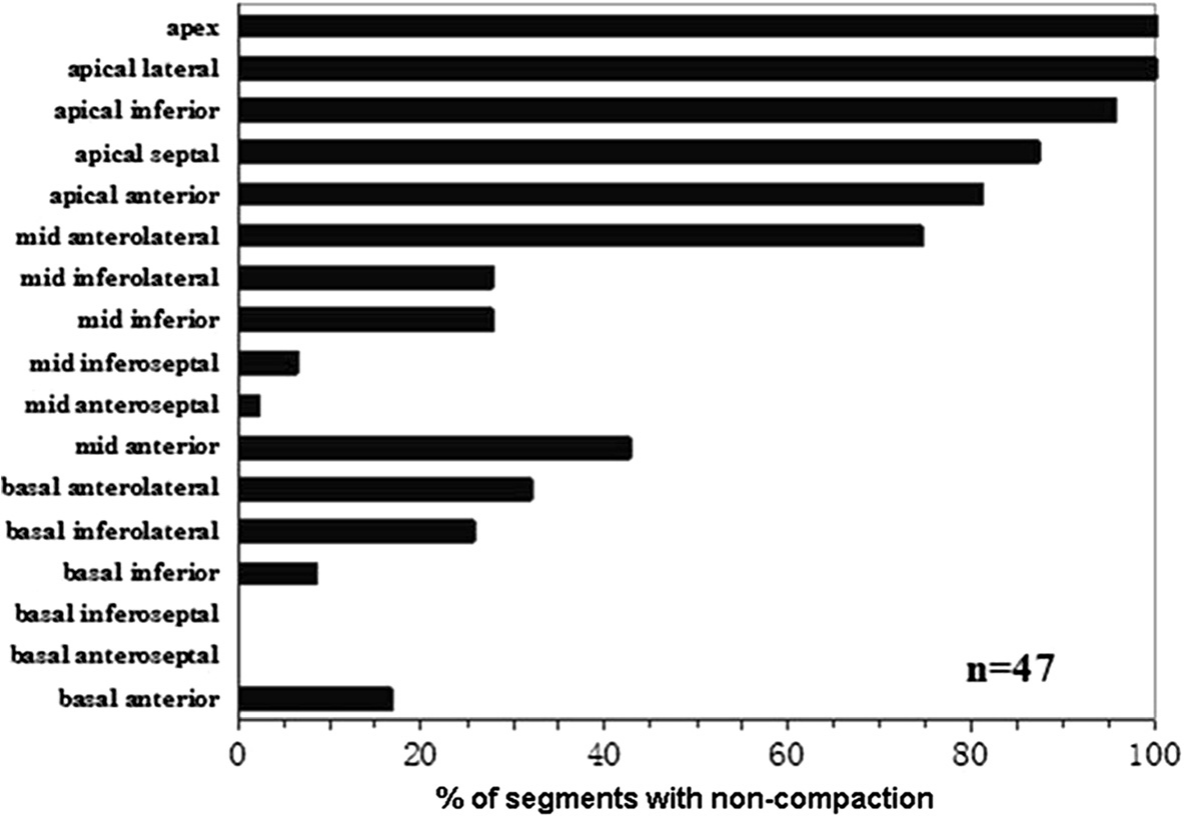
**Distribution of non-compaction. **The bars represent the percentage of segments with non-compaction in given segments.

Three hundred fifty (44%) LV segments were considered to fulfil the definition of non-compaction; the mean number of LV non-compacted segments per patient was 7.4 ± 2.5 and the NC:C was 3.2 ± 0.7. Non-compaction was most commonly noted in the apical segments in all patients. Basal-septal segment involvement was not noted in any patient (Figure 1).

**Figure 2:**
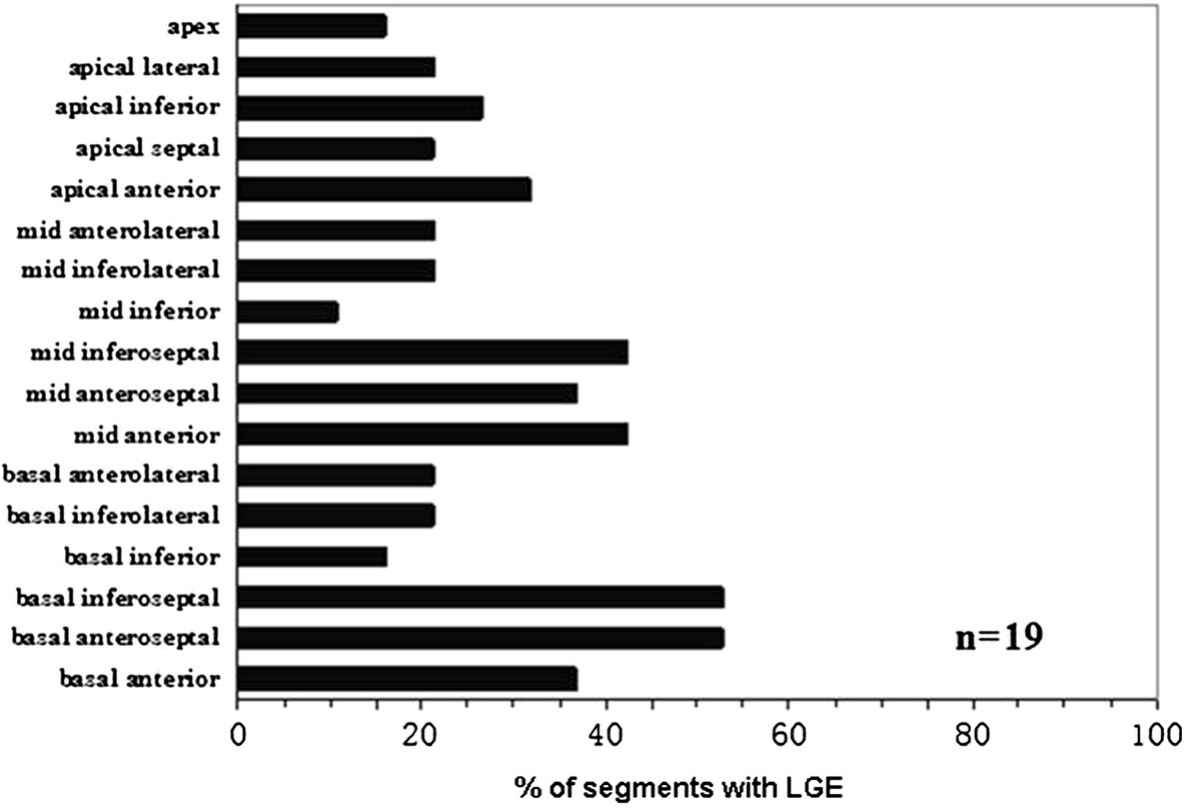
**Distribution of LGE. **The bars represent the percentage of segments with LGE in given segments. LGE=late gadolinium enhancement.

**Figure 3:**
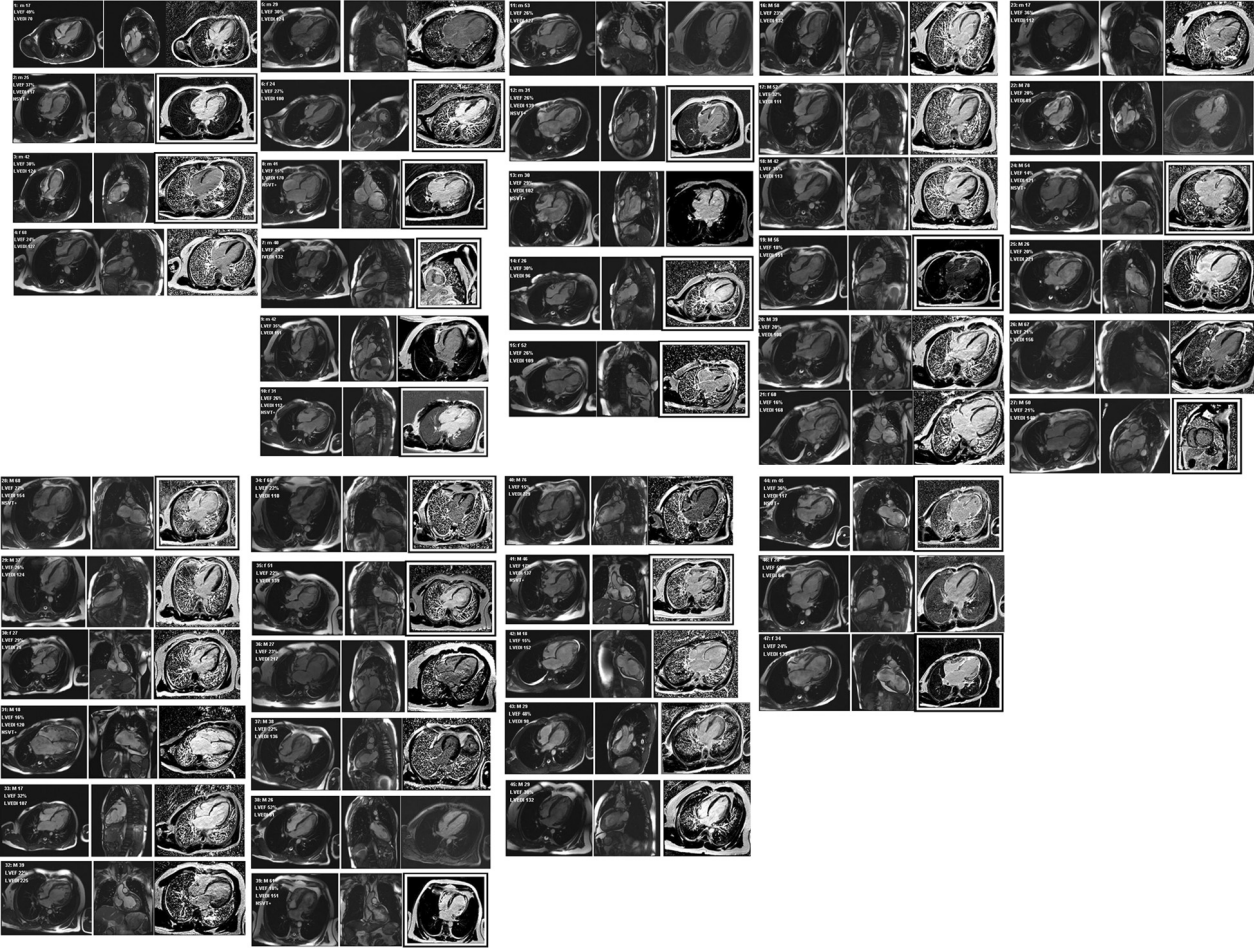
**CMR appearances of all 47 patients considered to meet criteria for LVNC. **For each patient, two SSFP images are shown and, on the right, one late gadolinium enhancement image, with a black and white frame if it is considered to show fibrosis. m = male or f = female, age given in years, LVEF = left ventricular ejection fraction. LVEDI = left ventricular end-diastolic volume index in ml/m^2^. NSVT + = non-sustained ventricular tachycardia documented.

LGE was evident in 19 (40%) of the 47 patients. LGE was present in all 17 segments, not only involved non-compacted segments but also normal segments of the heart. Totally, 90(28%) LV segments showed LGE. Seventy-two (80%) LGE (+) LV segments were located in compacted segments, most commonly in the ventricular septum (n = 40; 44%), while only 18 (20%) LGE (+) LV segments were in non-compacted segments (Figure 2). The mean number of LGE (+) segments per patient was 5.1 ± 3.6. The distribution of LGE was subendocardial (n = 5; 6%), mid-myocardial (n = 61; 68%), subepicardial (n = 10; 11%), and transmural (n = 14; 15%) in total of 90 LGE (+) segments (Figure 3).

## Discussion

LVNC has a wide spectrum of clinical and pathophysiological findings [16]. Clinical manifestations range from asymptomatic status to heart failure, arrhythmias, and systemic thromboemboli [17,18]. Echocardiographic criteria [1,7] for this disease based on the findings of a two-layered myocardium, with evidence of deep intertrabecular recesses filled with blood from the left ventricular cavity, along with a ratio of NC:C > 2.0:1.

CMR is becoming increasingly widely used in assessment of suspected LVNC. Petersen et al. [11] found that a ratio of NC:C >2.3 in diastole distinguished pathological non-compaction with values for sensitivity and specificity, and positive and negative predictions of 86, 99, 75, and 99%, respectively. Jacquier et al. [19] found that a trabeculated left ventricular mass above 20% of the global LV mass is highly sensitive and specific for the diagnosis of LVNC. However, Kawel et al. [20] reported that a NC:C > 2.3 could be found in 6% of a large cohort of subjects without clinically recognized cardiovascular pathology. Their data showed that the proposed criteria are unlikely to have adequate specificity.

In agreement with previous studies, we observed LGE in approximately 40% patients considered to meet criteria for LVNC. The possibly pathophysiological mechanisms leading to LGE in LVNC have not been determined. Jenni et al. [21] described the coronary microcirculatory dysfunction may possibly account for ischemia and fibrosis in non-compacted and compacted myocardial segments in LVNC. Coronary artery embolism may be considered to account for subendocardial or transmural LGE in young adults (such as cases 2, 6 and 15). Furthermore, most of the remaining LGE (-) studies would, in themselves, pass for those resulting from dilated cardiomyopathy and/or previous myocarditis.

Nucifora et al. [22] found the presence and extent of LGE were independently related to LVEF. In this present study, we compare the LGE (+) and LGE (-) patients, and find that LGE (+) group have lower LVEF, the p value of LVEF is close to significance (P = 0.06). The presence of LGE is associated with ventricular arrhythmias on ambulatory Holter ECG in patients meeting criteria for LVNC. The results need to be confirmed by further studies with a larger sample size.

### Limitations

Our study has a number of limitations. It was conducted at a single center with a relatively small sample size, and the diagnosis was based on CMR cine appearances in relation to clinical findings. However, not all patients had coronary artery disease ruled out by invasive coronary angiography. More thorough documentation of all previous investigations and findings would have been desirable. Clinical follow-up data were not available so the relationship between LGE and prognosis in LVNC patients remains unknown.

## Conclusions

In patients considered to meet criteria for LVNC, LGE distributions visible were strikingly heterogeneous with appearances potentially attributable to three or more distinct cardiomyopathic processes. This is in keeping with previous suggestions that the criteria may be of low specificity. Further work is needed to determine whether conditions such as dilated cardiomyopathy, previous myocardidtis or ischaemic heart disease increase the apparent depth of non-compact relative to compact myocardium.

## Competing interests

The authors declare that they have no competing interests.

## Authors’ contributions

JYW, SHZ and BCH conceived and designed the study. JYW drafted the manuscript. SHZ reviewed and edited the manuscript. MJL, GY and SLJ carried out images analyses. XJG and YJY were responsible for cases selected and analysis. All authors read and approved the final manuscript.

## Authors’ information

The study was supported by grant No. 81130029 from the key projects of National Natural Science Foundation of China and by grant No. 2009–1004 from Research Foundation of Capital Medical Development.
